# Antimicrobial resistance of *Escherichia coli* and *Salmonella* in raw retail table eggs in Lusaka, Zambia

**DOI:** 10.14202/vetworld.2020.2528-2533

**Published:** 2020-11-26

**Authors:** Munsanda Susan Kapena, John Bwalya Muma, Charles Miyanda Mubita, Musso Munyeme

**Affiliations:** 1 Department of Veterinary Services, Ministry of Fisheries and livestock, Chibombo District Veterinary Offices, Central Province, Zambia; 2 Department of Disease Control, School of Veterinary Medicine, University of Zambia, Lusaka, Zambia

**Keywords:** antimicrobial resistance, eggs, *Escherichia coli*, retail markets, *Salmonella*

## Abstract

**Background and Aim:**

Antimicrobial resistance (AMR) has risen as a serious cross-cutting global public health emergency. At the center of this emergency, foods of animal origin have particularly been singled out as possible drivers despite the paucity of information. This study has been formulated to provide answers to the identified critical gaps in the food safety industry and the public health sphere. In particular, this study was undertaken to investigate the AMR of *Escherichia coli* and *Salmonella* in raw retail table eggs in Lusaka, Zambia.

**Materials and Methods:**

Accordingly, a cross-sectional study to determine antibiotic susceptibility of *E. coli* and *Salmonella* from raw retail table eggs was undertaken. Standard bacteriological methods involving culture and phenotypic characterization were applied. A total of 1080 raw table eggs pooled into composite samples (five eggs per composite sample) translating into 216 distinct and independently identifiable compounded sample units were collected from randomly selected supermarkets and open markets over 4 months (August 2018-November 2018). The eggs were screened for the presence of *E. coli* and *Salmonella* within 24 h of sample collection by standard microbiological methods. The Kirby–Bauer disk diffusion technique was used for antimicrobial susceptibility testing using a panel of nine different antibiotics.

**Results:**

A total of 216 pooled egg samples were analyzed at two levels of contamination, (i) eggshell and (ii) egg content. From the eggshell, five compounded samples were positive for *Salmonella* spp. representing 2.31% (5/216), while 34.26% (74/216) were positive for *E. coli*. On the other hand, samples from egg contents were negative for *Salmonella* and *E. coli*. Eggshells were more likely to be contaminated by *E. coli* compared to the egg content (χ^2^=20.95, p<0.0001). Imipenem was 100% effective against *E. coli* isolates. With *Salmonella*, high resistance was seen in 80% against tetracycline (TE) and 60% to ampicillin (AMP). *E. coli* showed 94.6% resistance to colistin sulfate, 83.8% resistance to TE, and 59.5% resistance to AMP.

**Conclusion:**

Overall, this study has been able to demonstrate the presence of *E. coli* and *Salmonella* outside and inside table eggs in Zambia. It has also shown the resistance of identified isolates which poses a serious public health concern given the consumption patterns of these table eggs.

## Introduction

The last and half-century have seen massive development in antimicrobial resistance (AMR) in bacteria with food of animal origin contributing a considerably large proportion of these findings [1]. AMR has become a global public health emergency that is threatening to undermine decades of progress in the treatment of infectious diseases [2]. Antimicrobial is essential for the treatment of animals. Antimicrobials are essential for the treatment of sick animals, but even if used correctly, they may eventually lead to AMR [3]. The indiscriminate use of antimicrobials in animal farming is likely to accelerate the development of AMR in pathogens as well as in commensal organisms [4]. While this represents a potential hazard to humans, the great majority of resistant human pathogens, especially the more important ones, are unrelated to animal sources, further complicating the actual causal factors linked to the development of AMR in bacteria [5]. The worldwide increase in the use of antibiotics as an integral part of the poultry and livestock production industry to treat and prevent infectious bacterial diseases, as well as growth promoters, at subtherapeutic levels in feed has led to the problem of the development of bacterial antibiotic resistance during the past years [6]. In intensively reared food animals, antibiotics are administered for the therapeutic purpose and as antimicrobial growth promoters to the whole flock rather than individuals [7]. In as much as, it is beneficial to use antimicrobials in food animal production such as prevention and treatment of animal diseases, protection of humans against zoonosis, and enhancement of animal production, the risks of antimicrobials raise AMR concerns [8].

Poultry eggs are the most consumed food globally and they contribute significantly to a daily healthy diet of an average human being given their affordability coupled with their importance as a readily available source of protein [9]. In terms of nutritive value, the egg protein is a complete protein food as it has all nine of the essential amino acids (as well as all nine of the non-essential amino acids) [10]. Based on the essential amino acids, it provides, egg protein is second only to mother’s milk for human nutrition [6] but has an advantage on that they are less perishable than milk [11]. The consumption of eggs around the world in 2014 remained higher than a decade ago and the increase was particularly notable in developing countries where changing diets have been observed, whereby people are consuming a greater number of calories from protein sources such as poultry meat and eggs [9]. Even though eggs and their products have been found to contain high levels of cholesterol and so attract little patronage by adults, they remain a very important food for children [12]. In Zambia and other developing countries, rapid urbanization has resulted in an increased demand for livestock products which also includes eggs [13,14]. According to the Investor’s Guide on Poultry in Zambia, the egg consumption/capita/year stands at 66 eggs. The estimated total egg demand is 864,600,000, with the total annual egg production being 1,100,000,000, the estimated availability for export standing at 235,400,000, and the total annual egg consumption being at 1,000,000,000 [15]. The presence of highly nutritive substances in eggs in a conducive state creates an appropriate environment for the development of bacterial microbiota, including pathogenic bacteria, thus making eggs potential pathogens [7]. Contamination of eggs and egg products with microorganisms can affect egg quality, which may lead to spoilage and pathogen transmission [8]. Bacteria such as *Escherichia coli* and *Salmonella* have been identified as some of the organisms that easily colonizes eggs. Foodborne illnesses can lead to uncountable premature deaths, several health complications (typhoid fever and gastroenteritis), and massive losses in productivity. Diarrheal diseases are the most common illnesses implicated as a direct result of the consumption of contaminated food causing 550 million people to fall ill and 230,000 deaths every year[16]. Most *Salmonella* infections in humans’ results from the ingestion of contaminated poultry 3 and these infections are associated with the consumption of raw eggs and foods containing raw eggs[17]. It is undeniable that the rational use of antimicrobials plays a key role in the production of food animals and protecting public health, while irrational and irresponsible are likely to lead to AMR [8]. Antibiotic-resistant bacteria may reach humans (i) indirectly along the food chain through consumption of contaminated food or food-derived products and (ii) following direct contact with colonized/infected animals or biological substances such as blood, urine, feces, saliva, and semen among others[18]. The incidence of AMR is thus undoubtedly worse in developing countries, where humans interact intimately with animals and the environment, infectious disease rates are higher, regulations on antibiotic use, and the development, implementation, and monitoring of AMR prevention and containment measures are rare and frequently non-existent [10].

Poultry flocks are often raised under intensive conditions using large amounts of antimicrobials to prevent and to treat disease, as well as for growth promotion [4]. Therefore, circumstantial evidence indicates that there are unsanitary practices along the egg production chain that may be a contribution to bacterial contamination. Despite the increased demand in the production and consumption of table eggs, there is, however, inadequate information in Zambia on the microbiological quality of table eggs, foodborne pathogens and their AMR, and hygiene during production and processing. There is no information detailing the spectrum of bacteria specifically *E. coli* and *Salmonella* spp. found in raw retail eggs in Zambia, neither is there information relating to the proportions of these bacterial contaminations.

It is against this background that this study has been formulated to provide answers to the identified critical gaps in the food safety industry and the public health sphere. In particular, this study was undertaken to investigate the AMR of *E. coli* and *Salmonella* in raw retail table eggs in Lusaka, Zambia.

## Materials and Methods

### Ethical approval

During proposal development, both the Departmental Board and the School of Veterinary Medicine Board of Graduate studies approved this study after Ethical and Technical considerations.

### Study area and design

This study was a cross-sectional investigation of AMR of *E. coli* and *Salmonella* spp. in raw retail table eggs in Lusaka, Zambia, a comparative contamination assessment between egg content and eggshell. Study units were purposively selected within the capital city, given the high numbers of poultry suppliers of table eggs (retail eggs) and the highest concentration of retail shops (open markets and super chain stores). The primary sampling units were divided into the following strata as multistage stratified random sampling was employed; Strata 1: Eggs from open markets and Strata 2: Eggs from supermarkets/chain stores. The secondary sampling units were the individual eggs that were sampled. Inclusion criteria included retail eggs sold in open and supermarkets within Lusaka city, while exclusion criteria included retail eggs at the point of production, which includes both small scale and commercial poultry farms within and outside Lusaka city.

### Sample collection

Table eggs were purchased from selected markets in Lusaka throughout 4 months (August 2018-November 2018).

### Processing of samples

#### Eggshells

Sample collection from the eggshells involved dispensing 150 mL sterile buffered peptone water (BPW) was into sterile stomacher bags. An egg was then randomly picked from the tray using a pair of sterile forceps and placed into a sterile stomacher bag containing 150 mL sterile BPW. The stomacher bag and its contents were then shaken for 30 s, and the egg then removed using sterile forceps. The same procedure was used for the other four eggs using the same stomacher bag to make one sample. The eggshell rinsate was then incubated at 37°C for 24 h for pre-enrichment. The same procedure was applied for the remaining 216 eggshell samples.

#### Egg content

Sample collection from the egg content (albumin and yolk) involved disinfecting the outer surface of the eggs (the same eggs pooled as eggshell sample 1 in 4.5.1. above was used as egg content sample 1) by wiping using a surgical gauze soaked in 70% ethanol. The individual eggs were then cracked open using a sterile air of forceps and the contents poured into a sterile flask. The contents of sample 1 (egg content) were then homogenized and 50 mL dispensed into sterile falcon tubes. The same procedure was carried out to make the remaining 216 egg content samples.

### Determination of contamination of *Salmonella* species and *E. coli* in raw retail eggs (eggshells and egg contents) from both open markets and supermarkets

#### Isolation of Salmonella

About 1.0 mL of the pre-enriched eggshell rinsate was added to 9 mL enrichment Rappaport-Vassiliadis broth (HiMedia), which was then mixed using a vortex mixer before incubating at 42°C for 24 h. Similarly, 1 mL of the homogenized samples of egg content was also added to 9 mL Rappaport-Vassiliadis broth, which was then mixed before incubating at 42°C for 24 h. A loopful each of the enrichment broth of eggshell rinsate and egg content was then subcultured on Xylose Lysine Deoxycholate Agar (XLD, HiMedia) then incubated at 37°C for 24 h.

#### Isolation of E. coli

About 1.0 mL each of pre-enriched eggshell rinsate and egg content were added to 9 mL BPW, respectively, then mixed using a vortex before incubating at 37°C for 24 h. A loopful of the broth was subcultured on Eosin Methylene Blue agar (EMB agar) then incubated at 37°C for 24 h.

Suspect colonies from each sample were subcultured onto selective and non-selective agar to ensure that possible contaminants were absent. Further, isolates were inoculated onto composite media, including triple sugar iron agar, urea, lysine iron agar, Voges–Proskauer, methyl red (HiMedia, India), and SIM medium (HiMedia) [11]. All suspected *Salmonella* and *E. coli* colonies were further identified using Analytical Profile Index 20 E systems (bioMerieux SA, Marcy-1 Etoile, France). *E. coli* (ATCC 25922) was used as a quality control organism. Pure bacterial isolates were kept frozen at −80°C in 10% (v/v) glycerol peptone water broth for the evaluation of the antimicrobial sensitivity test.

### Antibiotic resistance evaluation of *E. coli* and *Salmonella* species associated with raw retail eggs (eggshells and egg contents) from both open markets and supermarkets

The antibiotic disk diffusion test using the Kirby–Bauer method was used [12]. A standard suspension of confirmed isolates of *Salmonella* species and *E. coli* was inoculated on the surface of Mueller-Hinton agar plates (Oxoid, UK). They were then tested for their susceptibility using filter paper disks (Oxoid) containing a specific concentration of nine antimicrobial agents as follows: Amoxicillin/clavulanic acid (AMC, 30 μg), ampicillin (AMP, 10 μg), cefotaxime (CTX, 30 μg), imipenem (IPM, 10 μg), chloramphenicol (C, 30 μg), nalidixic acid (NA, 30 μg), ciprofloxacin (CIP, 5 μg), colistin sulfate (CT, 10 μg), and tetracycline (TE, 30 μg) were then pressed on to the surface and incubated at 35°C overnight (24 h). After incubation, the zone of inhibition of growth of bacteria around each disk was measured and the susceptibility determined [13].

### Statistical analysis

The data were summarized and analyzed using STATA version 13.1 (StataCorp, College Station, Texas, USA). The test used to analyze the association between the occurrence of *Salmonella* and pathogenic *E.coli* was Chi-square test of independence. The level of significance was accepted at p≤0.05.

## Results

A total of 1080 raw table eggs were purchased from selected markets. The egg samples were pooled into composite samples (five eggs per composite sample), translating into 216 distinct samples (Table-1). Further, these eggs were grouped according to the source, namely: Open market (n=108) and closed market (n=108).

**Table-1:** Distribution of *Salmonella* and *Escherichia coli* based on source.

Source	No. of samples	Frequency of isolation of pathogens (%)			

		*Salmonella* spp.		*Escherichia coli*	
	
		Eggshell	Egg content	Eggshell	Egg content
Open market	108	3 (2.78)	0	32 (29.62)	0
Closed market	108	2 (1.85)	0	42 (38.88)	0
Total	216	5 (2.31)	0	74 (34.26)	0
p-value		0.651		0.000	

### Enumeration of *Salmonella* and *E. coli* from eggshells

Five (3.7%, 5/216) *Salmonella* and 74 (34.26%, 74/216) *E. coli* isolates were isolated and identified from 216 compounded samples (Table-1). Of the five, 3 (60%) isolates were from the eggs collected from the open market, while 2 (40%) were from a closed market. On the other hand, 32 (43.24%, 32/74) of *E. coli* were from open markets and 42 (56.76%, 42/74) were from a closed market. There were no significant differences in *Salmonella* contamination levels of eggshells (χ^2^=0.2047, p=0.651) between eggs from open market and closed markets. Significant differences in *E. coli* contamination levels on eggshells (χ^2^=20.95, p=0.000) were observed between the eggs from open market and closed markets.

### Enumeration of *Salmonella* and *E. coli* from egg content

Samples from egg contents were negative for *Salmonella* and *E. coli* (Table-1).

### AMR profiles

#### Overall AMR profiles for Salmonella and E. coli isolates

The overall distribution of AMR profiles against both *Salmonella* and *E. coli* is shown in Table-2. Both pathogens displayed high AMR profiles against TE (83.33%) and CT (83.33%) with low resistance with C (2.38%). IPM showed no resistance to any of the pathogens but 100% susceptibility (Table-2).

**Table-2:** Overall distribution of antimicrobial resistance across *Salmonella* spp. and *Escherichia coli* isolates.

Serial no.	Antibiotic	Frequency of antimicrobial phenotypic pattern (%)			

		*Salmonella* isolates (n=5)		*Escherichia coli* isolates (n=37)	
	
		Resistant	Susceptible	Resistant	Susceptible
1.	Cefotaxime	20	80	2.7	97.3
2.	Amoxicillin/clavulanic acid	0	60	18.9	48.7
3.	Nalidixic acid	20	40	32.4	29.7
4.	Ciprofloxacin	20	60	27.0	48.6
5.	Ampicillin	60	40	59.5	37.8
6.	Tetracycline	80	0	83.8	2.7
7.	Imipenem	0	80	0	100
8.	Chloramphenicol	20	40	0	59.5
9.	Colistin sulfate	-	-	94.6	0

NB: Intermediate resistance profiles are not included in the table. KEY: (-)=Not tested

#### AMR profiles for Salmonella isolates

The distribution of AMR profiles against only *Salmonella* isolates is shown in Table-2. *Salmonella* displayed high AMR profiles against TE (80%) and AMP (60%) with low resistance with C, while IPM showed no resistance but 100% susceptibility (Table-2).

#### AMR profiles for E. coli isolates

The distribution of AMR profiles against only *E. coli* isolates is shown in Table-2. *E. coli* displayed high AMR profiles against CT (94.59%), TE (83.8%), and AMP (59.5%) with low resistance with CTX (2.7% resistance), while C and IPM showed no resistance but 100% susceptibility (Table-2).

## Discussion

The present study revealed relatively high contamination of eggshells compared to the inner context (egg yolk and egg white) with a contamination rate of 2.31% (5/216) in the studied egg samples with *Salmonella* from eggshells. Further, this study revealed an overall contamination rate of 34.3% (74/216) in the studied egg samples with *E. coli* from eggshells. The results obtained in this study are similar to those obtained by Loongyai *et al*. [14], who detected *Salmonella* spp. at 5% of the samples of eggshells and *E. coli* at 35%, indicating a higher percentage of *E. coli* than *Salmonella*. Unfortunately, the authors were not able to explain the differences in this observation. From the biology of these microorganisms, during the competitive growth of a ray of bacteria, *E. coli* grows faster and also inhibits the growth of other organisms, including *Salmonella* [15]. However, the novelty in this study is that two postulations have been put forward. The first postulation is that the finding of *Salmonella* in the eggshell strongly intimates the possibility of fecal contamination during or after oviposition. The second postulation is that the finding of *E. coli* only on the shell, and none inside the egg, strongly intimates that *E. coli* contaminates the egg superficially, mainly through the fecal route by droppings, or during the hatching process through the cloacae. Further, *E. coli* may even contaminate the eggshell once it’s hatched in the outside environment of the chicken [14,19,20].

The results of this study further showed significantly higher contamination of egg surfaces with *E. coli* than with *Salmonella* at 34.3% and 2.31%, respectively. Another plausible explanation for this observation is the way that the host chickens shed these two bacteria; in chickens, *Salmonella* is shed intermittently and tends to be an intermediate intracellular pathogen in nature compared to *E. coli*, which can even be found in the open alimentary tract [16].

There was a comparatively higher proportion of *Salmonella* spp. in the market eggs, based on shell contamination. This may be due to surface contamination as a result of fecal presence in the environment on the one hand as well as during handling on the other hand. Other authors have indicated that contamination with *Salmonella* spp. can occur even during storage and transportation while the finding of contamination of egg contents points to the possible route of infection in the hen being a vertical process through the yolk or albumin [17].

Another key finding in this particular study was the prevalence of 80% and 83.3% resistance to *Salmonella* and *E. coli*, respectively. This is comparable to the prevalence of TE resistance in both *Salmonella* and *E. coli* from table eggs by other workers [18]. Overall resistance, AMP was the second most resistant antibiotic at a prevalence rate of 60% and 59.5% against *Salmonella* and *E. coli*, respectively. Out of the group of antimicrobials tested for susceptibility, only IPM was found to be 100% effective against *E. coli* isolates [21].

In this particular study, another important finding is that drugs commonly used in poultry such as TE showed high resistance rates compared to drugs rarely used such as IPM. Despite being beyond the scope of this present study, multi resistance was observed with *E. coli* isolates. In this study, *E. coli*, on its own, showed 94.6% resistance to CT, 83.8% TE, and 59.5% AMP. The present findings have serious public health implications. According to Rasheed *et al.*, [22], antibiotic resistance in E. coli is of particular concern because it is the most common Gram-negative pathogen in humans, the most common cause of urinary tract infections, a common cause of both community and hospital-acquired bacteremia, as well as a cause of diarrhea. It is important to note that besides, the resistant E. coli strains can transfer antibiotic resistance determinants not only to other strains of E. coli but also to other bacteria within the gastrointestinal tract and to acquire resistance from other organisms [23].

The resistance pattern of *Salmonella* spp. isolates in the present study was 60% AMP and 80% TE, which is higher compared to that obtained by Adesiyun, 2007, who reported zero resistance to TE. On the other hand, complete susceptibility of *Salmonella* spp. to TE has been reported [17]. This variation is most likely as a result of the differences in the application and use of such antibiotics as well as environmental factors such as antibiotic resistance selection pressure, which is directly linked to natural resistance genes, which is the scope for further study.

## Conclusion

Overall, this study has been able to demonstrate the presence of *E. coli* and *Salmonella* outside and inside table eggs in Zambia. It has also shown the presence of resistant isolates of *E. coli* and *Salmonella* outside and inside table eggs, which poses a serious public health concern given the consumption patterns of these table eggs.

## Authors’ Contributions

MSK: Contributed to the design, data collection, laboratory work, analysis of data, and drafting of the manuscript. JBM: Contributed to the design, supervision of laboratory work, and reviewing of the manuscript. CMM: Contributed to field studies and laboratory work, drafting and reviewing of the manuscript. MM: Contributed to the supervision of the project, acquisition of parts of the funds, writing of the manuscript, and important intellectual contribution. All authors have read and approved the final manuscript.
